# Social Determinants of Health and Dental Rehabilitation After Mandibular Reconstruction: A Scoping Review of an Evidence Gap

**DOI:** 10.3390/jcm15218318

**Published:** 2026-10-26

**Authors:** Ankit Juneja, Thomas J. Sorenson, Amitesh Verma, Alexis B. Jacobson, William West, Adam S. Jacobson, Jamie P. Levine

**Affiliations:** 1 Hansjorg Wyss Department of Plastic Surgery, NYU-Langone Health, 222 East 41st Street, New York, NY 10016, USA; ankit.juneja@nyulangone.org (A.J.); amitesh.verma@nyulangone.org (A.V.);; 2 Department of Otolaryngology-Head and Neck Surgery, NYU-Langone Health, New York, NY 10016, USA

**Keywords:** social determinants of health, dental restoration, mandible reconstruction, fibula free flap, scoping review

## Abstract

**Background**: Vascularized free flap reconstruction reliably restores mandibular continuity, but dental rehabilitation is delivered inconsistently. Because dental rehabilitation has been examined almost entirely through a clinical lens, we mapped whether and how social determinants of health (SDOH) have been examined in relation to its receipt after mandibular reconstruction. **Methods**: A scoping review following PRISMA-ScR searched PubMed/MEDLINE and Scopus from January 2000 through October 2026, supplemented by backward citation searching. Studies were charted by SDOH exposure and rehabilitation outcome. **Results**: Of 167 records screened, 14 were assessed in full text, and two met inclusion criteria. One cohort found race significantly associated with implant placement, with cost the most frequently reported barrier in that cohort; one case report framed prosthetic rehabilitation around affordability. One excluded cohort measured SDOH but reported only surgical outcomes. Across charted cohorts, completed rehabilitation ranged from 19% to 78% and implant placement from 27% to 38.5%, and contextual studies examined clinical determinants almost exclusively. **Conclusions**: Within the literature identified, the relationship between social circumstance and dental rehabilitation after mandibular reconstruction has been examined directly in only one small cohort and one case report. The financing divide is a plausible, testable mechanism, and cohort studies designed around SDOH exposures are needed.

## 1. Introduction

Segmental resection of the mandible for oral cavity malignancy, osteoradionecrosis, osteomyelitis, aggressive benign tumors, or trauma produces a discontinuity defect that impairs mastication, speech, swallowing, and facial form. Vascularized osteocutaneous free flap reconstruction, most often with the fibula, is the standard for restoring continuity and achieves excellent flap survival [1]. However, bony continuity is a means, not the end. The end is a functional, occluding dentition, and reaching it requires a second phase of care comprising osseointegrated implants, implant-supported or conventional prostheses, or, where reconstruction is not feasible, obturators.

This second phase is where reconstruction frequently stalls. A patient can leave the operating room with a well-contoured neomandible and often never receive the implants and prosthesis that would let them eat a normal diet or speak intelligibly [2,3,4]. We found that the clinical literature has extensively characterized how to rehabilitate the reconstructed mandible and how well implants survive once placed but has paid far less attention to who receives rehabilitation, and why some patients do not [5,6,7,8]. There are minimal data that describe whether social circumstances, rather than biology or surgical technique, determine the answer [9]. This scoping review maps whether, how, and with what study designs social determinants have been examined in relation to dental rehabilitation and argues that the resulting gap is both consequential and tractable.

For clarity, we use “dental rehabilitation” to mean the completed restoration of function (osseointegrated implants, an implant-supported or conventional prosthesis, or an obturator) rather than an intermediate step such as implant placement alone, and we use “social determinants of health” in its established sense: the non-medical conditions in which people are born, live, work, and access care, including insurance and dental benefit design, income, race and ethnicity, language, education, transportation, and area-level deprivation, rather than narrowly as insurance or cost. Race and ethnicity are treated here as social rather than biological categories: markers of differential exposure to structural, institutional, and access-related processes, including structural racism, rather than attributes that explain outcomes in themselves [10,11].

## 2. Methods

This review was not prospectively registered and was conducted with reference to the PRISMA extension for Scoping Reviews (PRISMA-ScR) [12]. Study selection is summarized with a PRISMA flow diagram in Figure 1. PubMed/MEDLINE and Scopus were searched on 9 October 2026 for English-language literature published from January 2000 onward, combining title and abstract terms for mandibular resection or reconstruction (mandibulectomy, mandibular resection, mandibular reconstruction, jaw reconstruction, fibula free flap, osteocutaneous flap, free flap, reconstruction), dental rehabilitation (dental implant, oral rehabilitation, dental rehabilitation, denture, obturator), and social determinants (social determinants of health, socioeconomic status, insurance, Medicaid, race, ethnicity, disparity, area deprivation, social vulnerability, cost, affordability, out-of-pocket, income, travel distance). The Scopus strategy was adapted from the PubMed strategy using equivalent title and abstract field tags. The complete search strings, date of execution, and filters applied are provided in Appendix A. Backward citation searching was performed using the reference lists of included articles and relevant reviews. Eligible records were peer-reviewed, English-language reports of original research in adults after segmental mandibular reconstruction that addressed social factors in relation to the receipt, timing, or accessibility of dental rehabilitation. Case reports addressing affordability or accessibility of prosthetic care were also considered. Consistent with scoping review methodology, which aims to map the extent and nature of available evidence rather than to estimate effects, sources were eligible regardless of design [13]. Case reports were, therefore, charted as descriptive evidence of how social factors have been considered in rehabilitation and were not interpreted as evidence of an association between a social exposure and rehabilitation receipt. Review articles, conference abstracts, and the unpublished or gray literature were excluded from the included-study total. Studies reporting social exposures but surgical outcomes alone, and literature on clinical or technical determinants, were discussed as background and were not counted among the included studies. Two reviewers (T.J.S. and A.J.) independently screened titles and abstracts and subsequently assessed full texts for eligibility. Disagreements were resolved through a third reviewer (A.V.). T.J.S. abstracted population, exposure, and rehabilitation outcome data into a structured chart. Included studies were summarized descriptively by study design, population, social exposure examined, rehabilitation outcome, and principal finding (Table 1). To situate these findings, studies identified during screening and backward citation searching that measured social exposures without a dental rehabilitation outcome, or that reported rehabilitation outcomes without examining a social exposure, were charted separately as contextual literature (Table 2) and were not counted among the included studies. For synthesis, evidence was classified into three tiers: direct evidence, comprising studies that addressed a social factor in relation to dental rehabilitation (Table 1); adjacent evidence, comprising studies that measured social determinants in the relevant surgical population without a dental rehabilitation outcome (Table 2, Panel A); and contextual literature, comprising clinical studies of rehabilitation that examined no social exposure (Table 2, Panel B) and the broader literature on head and neck cancer disparities and dental coverage policy. Implant receipt and prosthesis delivery were distinguished when reported. No formal risk-of-bias assessment or meta-analysis was undertaken because the purpose was to map the available evidence rather than estimate a pooled effect.

## 3. Results

The database searches identified 228 records (PubMed/MEDLINE, n = 110; Scopus, n = 118), and backward citation searching identified 12 additional records. After removal of 73 duplicates, 167 records were screened by title and abstract against the eligibility criteria above, and 153 were excluded. Fourteen reports were assessed in full text, of which 12 were excluded because they did not examine a social exposure in relation to a dental rehabilitation outcome (Figure 1).

Two studies met full inclusion criteria and constitute the included evidence for this review (Table 1): a retrospective cohort by Peacock and Ji [9] and a case report by Dosumu and colleagues [14]. The first, a 52-patient retrospective cohort of patients resected for benign mandibular neoplasms, measured race against dental implant receipt. Twenty patients (38.5%) received implants, and race was significantly associated with implant receipt; only one of eleven Black patients (9.1%) received implants. Ten of the twenty implanted patients proceeded to a prosthesis, corresponding to completed rehabilitation in 19% of the cohort, and cost was the reason most frequently reported by participants for not receiving implants (37.5%). Because this association concerns implant placement, it addresses an intermediate step rather than completed rehabilitation as defined in this review. The second, a case report, described interim prosthetic rehabilitation in which affordability determined the prosthetic approach, and recommended that national health insurance cover such prostheses [14]. As a single descriptive case, it documents that financing has been considered in rehabilitation decisions but provides no evidence regarding association. Among the excluded reports, a 131-patient cohort that measured county-level social vulnerability, travel distance, race, language, and payer in patients undergoing osteocutaneous free flap reconstruction for mandibular malignancy met the population and exposure criteria but reported only surgical outcomes rather than dental rehabilitation; it is charted separately as adjacent evidence rather than as an included study [15].

No identified study modeled a social determinant against dental rehabilitation receipt using a validated area-level deprivation measure, such as the Area Deprivation Index or Social Vulnerability Index, or a multivariable framework accounting for clinical confounders. Across the included and contextual cohorts, completed rehabilitation, defined as delivery of a functional prosthesis, ranged from 19% to 78%, and implant placement alone ranged from 27% to 38.5% (Table 1 and Table 2). A highly selected series of 13 patients undergoing immediate implant placement, in which every patient received a prosthesis, was not included in this range, and a systematic review of 36 studies reported a pooled prosthetic rehabilitation rate of 51.6%. The contextual studies examined clinical or technical determinants almost exclusively; only the two included records addressed a social exposure in relation to rehabilitation.

## 4. Discussion

### 4.1. Dental Rehabilitation as the Functional Endpoint of Reconstruction

Restoration of occlusion and oral function is widely framed as the ultimate objective of jaw reconstruction, not an optional add-on [18]. Implant-supported prostheses overcome the limitations of tissue-borne dentures on reconstructed, often insensate and irradiated soft tissue, and are associated with improved oral function and quality of life [20]. When rehabilitation is completed, patients regain mastication, intelligible speech, and aesthetic support; when it is not, the reconstruction delivers a fraction of its potential benefit. The equity stakes of dental rehabilitation are, therefore, not only cosmetic; they are the difference between an anatomically successful operation and a functionally successful one [21,22].

### 4.2. The Reconstruction–Rehabilitation Gap

Multiple analyses show that a substantial share of reconstructed patients never complete rehabilitation, and that even successful pathways are slow. A 2024 systematic review of 36 studies estimated the pooled dental prosthetic rehabilitation rate after fibula free flap jaw reconstruction at roughly 52% (ranging from about 38% to 55% by benign versus malignant indication) [16]. Consistent with this, fewer than 40% of patients in a benign neoplasm cohort went on to receive implants, and only half of those proceeded to a prosthesis [9].

The pathway is also protracted. In a cohort of 52 patients reconstructed with vascularized bone flaps, rehabilitation was reached in 62% of an immediate implant group versus 78% of a delayed group, with a striking difference in timeline. There was a median of 150 days to prosthesis in the immediate pathway versus 843 days in the delayed pathway [17]. A comparison of prefabricated versus conventional bone-driven, delayed strategies reported mean time to occlusal rehabilitation of roughly 12 versus 60 months [18]. Immediate rehabilitation at the time of reconstruction, though feasible even in malignancy, remains uncommon in practice [19,23,24,25].

Two themes emerge. Non-receipt is common, affecting roughly half of patients. Further, the conventional delayed pathway that spans from months to years creates a long corridor during which patients can be lost to rehabilitation, including recurrence, death, attrition, or the accumulating friction of cost and access. Any factor that raises that friction differentially across social groups would be expected to produce inequity in the functional outcome of reconstruction.

### 4.3. The Traditional Lens

The dominant framing of “who gets rehabilitated” is clinical. Radiotherapy is the most consistently cited biological barrier: pooled data show implant survival falling from about 90–93% in non-irradiated bone to roughly 76% in irradiated bone, with survival as low as 38% when implants are placed within 12 months of radiotherapy, recovering toward 77% by 24 months [16,26]. Adequate flap bone height, favorable defect geometry, disease control, and comorbidity burden all bear on candidacy.

In parallel, technique has advanced specifically to close the rehabilitation gap: the double-barrel fibula to gain vertical height, with systematic review evidence of high flap survival (~98%) and low implant failure (~1.7%) [27]; virtual surgical planning; and immediate and prefabricated approaches that compress the timeline [18,19]. This literature is mature and valuable, but it implicitly treats non-receipt as a matter of biology and technique. It optimizes candidacy and success conditional on the patient reaching the dental chair; it does not ask which patients reach the chair at all.

This binary is not exhaustive, however. Non-receipt need not be either a biological or technical failure on one hand or a marker of social disadvantage on the other: health system characteristics, such as whether a prosthodontist is embedded in the cancer center or how surgical and dental teams coordinate care, and psychosocial factors not reducible to social position, such as treatment exhaustion, symptom burden, depression, or a patient’s own weighing of whether a prosthesis is worth pursuing, plausibly shape rehabilitation receipt as well. We focus below on social determinants because that is the axis this review set out to map and because, as detailed below, it has rarely been examined in the studies identified by this review; the near-absence of evidence on system-level and psychosocial explanations is a parallel gap that merits its own inquiry.

### 4.4. The Missing Lens

The step from “reconstructed” to “rehabilitated” is an access-and-utilization transition, a point at which social determinants are known to shape care in other settings, yet few of the studies identified by this review examined it through that lens. In what follows, we distinguish direct evidence (Table 1), adjacent evidence (Table 2, Panel A), and the contextual literature; only the first bears on the review question itself. The direct evidence consists principally of Peacock & Ji: in their benign neoplasm cohort, Black patients were the least likely to receive implants, and cost was the most cited reason for not proceeding [9]. The study’s own data point toward an access-related interpretation of this racial gradient: among patients who did not receive implants, cost was cited as the reason by five of ten Black patients (50%) compared with three of eighteen White patients (16.7%) [9]. The racial difference, therefore, appears to operate at least in part through financial barriers rather than through any characteristic of race itself, although the study was not designed to test this pathway formally. This is a small, single-center, benign disease study, but it is the only study identified by this review that measured a social exposure against dental-rehabilitation receipt after mandibular resection.

Some caution is warranted in weighing this single study, however. The report does not fully specify how long after resection patients’ reasons for not proceeding were ascertained, and cost was not the only reason cited: of 32 patients without implants, 12 (37.5%) cited cost or lack of insurance coverage, 10 (31.3%) did not want implants, and eight (25.0%) were lost to follow-up. Reasons recorded years removed from surgery are vulnerable to recall and rationalization, and a “disinterest” response may itself be downstream of the very barriers reviewed here rather than independent of them.

The closest adjacent work examined SDOH in patients undergoing osteocutaneous free flap reconstruction for mandibular malignancy, which is the right population and right exposures, but its outcomes were surgical, not dental rehabilitation, and within a well-resourced tertiary center, it found largely equitable surgical outcomes [15]. It, therefore, establishes the population and the plausibility of studying SDOH here while leaving the rehabilitation question open. Beyond these, the direct evidence thins to case reports of prosthetic rehabilitation framed around affordability in low-resource settings [14].

Two features of this literature deepen the concern. First, the Bhethanabotla et al. study measured county-level social vulnerability, travel distance, race, language, and payer in 131 patients and found surgical outcomes to be equitable; its only significant social finding, which was that non-minoritized patients travelled farther to reach care, may reflect referral catchment rather than an access barrier. Equity at the operative step, however, cannot be assumed to extend to the prosthetic one, and these patients were not followed to rehabilitation [15]. Second, the cohorts that report non-receipt attribute it almost entirely to causes that appear straightforward in the medical record: death and recurrence in a ten-year series in which 27% received implants and 20% completed rehabilitation, and patient motivation in a cross-sectional cohort in which complete oral rehabilitation reached 23.7% [2,4]. It is important to note that motivation is not a fixed patient trait. It is shaped by out-of-pocket cost, travel burden, time away from work, and whether a prosthodontist is embedded in the cancer center. Reasons recorded in clinical language may, therefore, conceal social ones, a possibility that only a study designed around SDOH could evaluate (Table 2).

The contextual literature from head and neck oncology makes it plausible, though untested, that social gradients operate at the rehabilitation step [28]. Disparities by race, socioeconomic status, and insurance are best documented in head and neck cancer among all otolaryngologic conditions, with differences in survival, recurrence, and mortality [29], and socioeconomic deprivation is associated with materially worse spatial access to treatment, i.e., the most deprived patients travel nearly twice as far to care [30]. However, plausibility is not evidence, and the specific question remains unanswered: after the mandible is rebuilt, do social circumstances determine whether it is made functional?

### 4.5. A Proposed Mechanism

There is a structural reason to suspect inequity at this step, and one that clinical studies are not designed to detect. Reconstruction is financed as medical care; dental rehabilitation frequently falls into the “dental” category that traditional Medicare has excluded and that many commercial and Medicaid plans cover sparingly or carve out entirely [31]. The consequence is a discontinuity in coverage that maps onto the discontinuity in care: the same patient whose resection and free flap are fully covered may face high out-of-pocket costs for the implants and prosthesis that make the reconstruction usable. Commentators have increasingly named this shortfall of restorative dental care for head-and-neck cancer patients as a systemic failure requiring policy attention [31,32].

The evidence that cost operates as a barrier at this step is, however, limited to the self-reported reasons of participants in a single small cohort with benign disease, in which patient disinterest was reported nearly as often as cost [9]. It does not establish that cost is the principal determinant of non-receipt in general, and the relative contributions of financial, logistical, system-level, and psychosocial factors remain unknown. What the coverage discontinuity offers is a specific and testable hypothesis. If financing matters, receipt of rehabilitation should track insurance type, dental benefit design, income, and the geographic distribution of prosthodontic care, and any resulting disparity would not be closed by better flaps alone. Testing this hypothesis against the alternatives is the measurable heart of the gap.

### 4.6. Why the Gap Has Persisted

Part of the reason this question has so rarely been addressed in the literature identified here is that dental rehabilitation is nearly invisible in the data sources reconstructive research relies on. Cancer registries and national surgical databases capture resection, flap type, and adjuvant therapy but not implant placement or prosthesis delivery. In administrative claims, dental procedures are coded in a separate nomenclature and adjudicated by separate carriers, so a patient’s implants may leave no trace in the medical claims that record their free flap. Institutions reproduce the same divide: the operative record sits in the medical chart while the prosthodontic record often sits in a dental practice management system, sometimes at another site and frequently unlinked. The consequence is that a well-designed study can fail to observe its own outcome. Any serious attempt at this question must, therefore, begin with outcome ascertainment, through deliberate linkage of surgical and dental records or prospective capture, rather than assuming rehabilitation status can be extracted retrospectively from existing fields. This is tractable but not trivial, and it likely contributes to why plausibility has outrun evidence.

### 4.7. Why This Matters and a Research Agenda

Dental rehabilitation converts an anatomically successful reconstruction into a functionally successful one. If its receipt is patterned by social circumstance, then technically equitable reconstructive surgery may deliver unequal functional outcomes. Testing this requires studies designed around social determinants and dental rehabilitation rather than surgical outcomes. A single-institution retrospective cohort at a high-volume center is a natural first step and, on its own, would roughly double the direct evidence identified by this review.

These questions benefit from an explicit theoretical scaffold rather than an atheoretical list of exposures. Andersen’s Behavioral Model of Health Services Use, which organizes predisposing (demographic and social–structural), enabling (income, insurance, access to a prosthodontist), and need (perceived and clinical) factors, maps directly onto the reconstruction-to-rehabilitation transition and would let a study distinguish whether non-receipt reflects a structural barrier, a resource barrier, or a needs-assessment gap [33]. The NIMHD Research Framework, which crosses levels of influence (individual, interpersonal, community, societal) with domains (biological, behavioral, physical/built environment, sociocultural environment, healthcare system), offers a complementary way to organize which exposures are captured and at what level [34]. A single-institution retrospective cohort, our own proposed first step, is also necessarily bounded by what the chart contains: payer, race/ethnicity, language, and area-level indices are extractable, but constructs central to the frameworks above, including health literacy, transportation, housing stability, instrumental and emotional social support, perceived financial or nutritional insufficiency, and work accommodations, are not. Closing that gap ultimately requires prospective data collection, whether through patient-reported measures embedded in a cohort or a mixed methods design pairing chart-derived exposures with qualitative interviews on the reasons for non-receipt.

Priorities should be:

1. Measure SDOH as exposures against rehabilitation receipt at the individual level (payer, and specifically dental coverage; race and ethnicity; language; social support) and the area level (Area Deprivation Index, Social Vulnerability Index, rurality, travel distance). Race and ethnicity should be analyzed as markers of structural and institutional exposure, alongside the factors they may index, such as insurance, area deprivation, and proximity to prosthodontic care, so that any disparity can be traced to a modifiable mechanism rather than attributed to race itself.
2. Model time to rehabilitation with competing risks methods (death, recurrence), not naive time-to-event given the multi-year pathway.
3. Use complementary designs including institutional cohorts (rich clinical and pathway detail) and large administrative or registry datasets (statistical power and area-level linkage) to provide answers to the full scope of questions.
4. Standardize the outcome. Completed rehabilitation ranges from 19% to 78% across the cohorts in Table 1 and Table 2, and much of that spread is definitional rather than real: some studies count implant placement, other studies count prosthesis delivery, and some count function at last follow-up. Equity research needs a stable denominator and one explicit endpoint. We would favor that being “functional prosthesis” with “placement of implants” retained as an intermediate step.

### 4.8. Strengths and Limitations

This review has several strengths. It was conducted with reference to PRISMA-ScR, searched two databases with fully reported strategies, defined dental rehabilitation and social determinants of health explicitly before charting, and used independent dual screening of titles, abstracts, and full texts with adjudication by a third reviewer. Backward citation searching supplemented the database searches, and studies meeting the inclusion criteria were charted separately from the contextual clinical literature so that the extent of the evidence gap is shown directly rather than inferred. Several limitations should also be acknowledged. First, only two databases, PubMed/MEDLINE and Scopus, were searched; Embase, Web of Science, and CINAHL were not, and relevant dental, prosthodontic, or health services reports indexed only in those sources may have been missed. Second, the search was restricted to peer-reviewed, English-language reports, and conference abstracts and the gray literature were excluded. This is particularly relevant because work on the affordability of prosthetic care may originate in settings that publish in other languages or outside indexed journals. Third, data charting was performed by a single reviewer without independent verification, which increases the risk of extraction error, although the charted variables were few and largely descriptive. Fourth, the review protocol was not prospectively registered. Fifth, only two studies met inclusion criteria, one of which was a single descriptive case report that cannot inform association, and outcome definitions varied across both included and contextual studies. Consistent with scoping review methodology, no formal risk-of-bias assessment was undertaken, so our findings describe the extent of the evidence rather than its quality. Finally, clinical cohorts that recorded race, insurance, or other social variables only as descriptive covariates, without analyzing them as determinants of rehabilitation, may not have been captured by our search terms. Taken together, these limitations mean that the near-absence of direct evidence should be read as a strong signal of a research gap rather than as proof that no relevant data exist.

## 5. Conclusions

The clinical literature has extensively characterized how to rehabilitate the reconstructed mandible and how to maintain implants once placed. Among the studies identified by this review, however, the question of who receives that rehabilitation, and why, has been examined directly in only one small single-center cohort and one case report, and this evidence is insufficient to establish whether social circumstance shapes access to it. Given a plausible and testable structural hypothesis, the equity of the reconstruction-to-rehabilitation transition remains an open question that well-designed cohort studies should address.

## Figures and Tables

**Figure 1:**
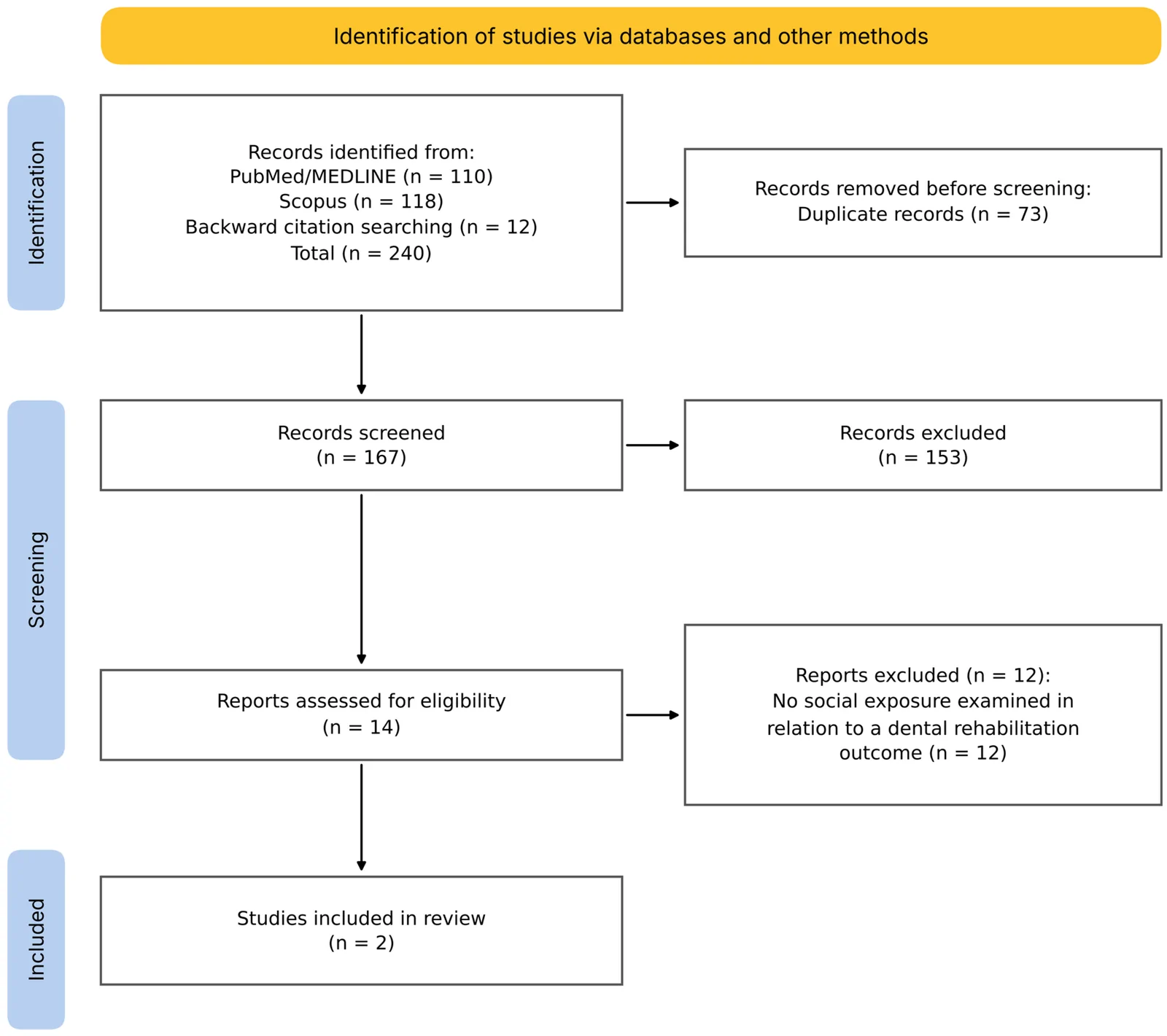
PRISMA 2020 flow diagram of study selection. The two included studies are presented in Table 1. Contextual studies charted in Table 2 did not meet inclusion criteria and are not counted among the included studies.

**Table 1:** Studies meeting inclusion criteria: social determinants examined in relation to dental rehabilitation after mandibular reconstruction.

Study (Year)	Design, n, Population	Social Exposure Examined	Rehabilitation Outcome	Principal Finding Related to SDOH
Peacock & Ji (2017) [9]	Retrospective cohort; n = 52; resection for benign mandibular neoplasm	Race; cost and insurance coverage as patient-reported reasons for non-receipt	Implants 20 of 52 (38.5%); prosthesis 10 of 52 (19.2%)	Implant receipt lowest among Black patients; cost most cited barrier, with patient disinterest nearly as common; no multivariable adjustment
Dosumu et al. (2012) [14]	Case report; n = 1; postmandibulectomy defect	Affordability and accessibility of prosthetic care (descriptive)	Interim prosthetic rehabilitation with a single-piece acrylic denture	Affordability determined the prosthetic approach; authors recommended national insurance coverage of interim prostheses. Descriptive only; no exposure–outcome comparison possible

SDOH, social determinants of health.

**Table 2:** Contextual literature not meeting inclusion criteria. Panel A. Adjacent evidence: social determinants measured without a dental rehabilitation outcome. Panel B. Contextual literature: dental rehabilitation outcomes reported without examination of a social exposure.

Study (Year)	Design, n, Population	Outcome Reported	Rehabilitation Received	Determinants Examined
Panel A. Adjacent evidence				
Bhethanabotla et al. (2025) [15]	Retrospective cohort; n = 131; osteocutaneous free flap for mandibular malignancy	Surgical outcomes only	Not reported	Race, language, insurance, county Social Vulnerability Index, travel distance
Panel B. Contextual literature				
Nham et al. (2024) [16]	Systematic review; 36 observational studies; fibula free flap jaw reconstruction	Dental prosthetic rehabilitation rate	51.6% pooled (38–55% by indication)	Radiotherapy timing, prosthesis type, virtual surgical planning
Ritschl et al. (2024) [2]	Cross-sectional; n = 59; fibula free flap mandibular reconstruction	Implant placement; prosthetic restoration; quality of life	Implants 37.3%; complete oral rehabilitation 23.7%	Implant survival, satisfaction, patient motivation
Oldén et al. (2023) [4]	Retrospective cohort; n = 41 (42 flaps); fibula free flap mandibular reconstruction	Implant placement; completed rehabilitation	Implants 27%; completed rehabilitation 20%	Radiotherapy; death and recurrence cited as reasons for non-receipt
Lodders et al. (2021) [3]	Retrospective cohort; 161 fibula free flap reconstructions; head and neck cancer	Implant-based rehabilitation started and completed	Started in 27.9%; 37 completed, 29 functional	Radiotherapy, tobacco use, body mass index
Tumuluri et al. (2023) [17]	Retrospective cohort; n = 52; vascularized bone flap mandibular reconstruction	Rehabilitation achieved; time to prosthesis	62% immediate vs. 78% delayed; median 150 vs. 843 days	Timing of implant placement
Tabet et al. (2024) [18]	Cohort; n = 21 (9 prefabricated, 12 delayed); benign or previously treated malignant jaw tumor	Time to occlusal rehabilitation	Mean 12.1 vs. 60.4 months	Reconstructive technique
Jelmini et al. (2024) [19]	Retrospective cohort; n = 13; immediate fibula free flap with implants for malignancy	Immediate prosthesis delivery; implant success	Prosthesis delivered in all (highly selected)	Implant success, delay to radiotherapy

Rehabilitation is defined differently across studies, so rates are not directly comparable. Studies in this table did not meet inclusion criteria and are not counted among the included studies in Figure 1.

## Data Availability

The original contributions presented in this work are included in the article and Supplementary Materials. Further inquiries can be directed to the corresponding author.

## Supplementary Materials

The following supporting information can be downloaded at: https://www.mdpi.com/article/10.3390/jcm15218318/s1, File S1. Scoping Reviews (PRISMA-ScR) Checklist.

## Appendix A. Search Strategies

PubMed/MEDLINE. Date searched: 9 October 2026. Records retrieved: 110.

(mandibulectomy[tiab] OR “mandibular resection”[tiab] OR “mandibular reconstruction”[tiab] OR “jaw reconstruction”[tiab] OR “fibula free flap”[tiab] OR “fibular free flap”[tiab] OR osteocutaneous[tiab] OR “free flap”[tiab] OR “reconstruction”[tiab]) AND (“dental implant*”[tiab] OR “oral rehabilitation”[tiab] OR “dental rehabilitation”[tiab] OR denture*[tiab] OR obturator*[tiab]) AND (“social determinant*”[tiab] OR socioeconomic[tiab] OR insurance[tiab] OR uninsured[tiab] OR Medicaid[tiab] OR race[tiab] OR racial[tiab] OR ethnicity[tiab] OR disparit*[tiab] OR “area deprivation”[tiab] OR “social vulnerability”[tiab] OR cost[tiab] OR costs[tiab] OR afford*[tiab] OR “out-of-pocket”[tiab] OR income[tiab] OR “travel distance”[tiab] OR “disparity”[tiab])

Scopus. Date searched: 9 October 2026. Records retrieved: 118.

TITLE-ABS(mandibulectomy OR “mandibular resection” OR “mandibular reconstruction” OR “jaw reconstruction” OR “fibula free flap” OR “fibular free flap” OR osteocutaneous OR “free flap” OR reconstruction) AND TITLE-ABS(“dental implant*” OR “oral rehabilitation” OR “dental rehabilitation” OR denture* OR obturator*) AND TITLE-ABS(“social determinant*” OR socioeconomic OR insurance OR uninsured OR medicaid OR race OR racial OR ethnicity OR disparit* OR “area deprivation” OR “social vulnerability” OR cost OR costs OR afford* OR “out-of-pocket” OR income OR “travel distance”) AND PUBYEAR > 1999 AND LANGUAGE(english) AND NOT DOCTYPE(cp)
